# New therapeutics to modulate mitochondrial dynamics and mitophagy in cardiac diseases

**DOI:** 10.1007/s00109-015-1256-4

**Published:** 2015-02-05

**Authors:** Marie-Hélène Disatnik, Sunhee Hwang, Julio C. B. Ferreira, Daria Mochly-Rosen

**Affiliations:** 1 Department of Chemical and Systems Biology, Stanford University School of Medicine, Stanford, CA 94305 USA; 2 Department of Anatomy, Institute of Biomedical Sciences, University of São Paulo, São Paulo, 05508-000 Brazil; 3 Department of Chemical and Systems Biology, Room 3145A, CCSR, Stanford University School of Medicine, 269 Campus Drive, Stanford, CA 94305-5174 USA

**Keywords:** Cardiac disease, Mitochondria, Mitophagy, Autophagy, Fission, Fusion, Mitochondrial dynamics, Heart

## Abstract

The processes that control the number and shape of the mitochondria (mitochondrial dynamics) and the removal of damaged mitochondria (mitophagy) have been the subject of intense research. Recent work indicates that these processes may contribute to the pathology associated with cardiac diseases. This review describes some of the key proteins that regulate these processes and their potential as therapeutic targets for cardiac diseases.

## Introduction

### Mitochondria-complex organelles

Mitochondria are central for cell fate. They are the major source of energy (ATP) and reducing power (NADH and NADPH). They are also a major cause for cell destruction by producing reactive oxygen species (ROS) and triggering several processes that lead to programmed cell death or apoptosis. It is therefore not surprising that mitochondrial dysfunction contributes to many diseases, including cardiovascular [1], neurological [2], and metabolic diseases as well as aging [3]. The first high-resolution micrograph of a mitochondrion in 1952 showed that this organelle has a double membrane, with the inner membrane forming invaginations (called cristae) into the mitochondrial matrix [4]. Mitochondria form a highly dynamic network that varies in size and shape in different cell types. The mitochondria in cardiac myocytes, however, are quite uniformly sized and nested between the contractile elements [1], whereas in other tissues (e.g., endothelial cells), the mitochondria form a perinuclear network that extends to the cell periphery [5]. Therefore, unique processes may govern mitochondrial shape and elimination in cardiac myocytes as compared with those in other cell types.

### Proteins that mediate mitochondrial dynamics

Mitochondrial size and number are tightly regulated by fusion and fission. These processes, which collectively are called mitochondrial dynamics, are orchestrated by a family of large GTPases and their respective adaptor proteins in the mitochondria. Mitochondrial fission is triggered by dynamin-related protein 1 (Drp1) [6–8]. Studies from yeast to mammalian cells have demonstrated that upon activation, Drp1 translocates from the cytosol to the outer mitochondrial membrane and binds to mitochondrial fission protein 1 (Fis1) [7, 9, 10]. Following activation, Drp1 oligomerizes and its GTPase activity increases, which results in constriction of the mitochondria at the scission sites and drives mitochondrial fission [7]. Excessive fission and mitochondrial fragmentation are increased in cells overexpressing Fis1 resulting in cell apoptosis [11]. In contrast, expression of a GTPase-defective dominant negative mutant of Drp1, Drp1(K38A), leads to inhibition of mitochondrial fission [12]. A number of other adaptor proteins have been found to recruit Drp1 to the mitochondrial membrane in mammalian cells. These include mitochondrial fission factor (MFF), which binds Drp1 independently of Fis1 [13] and MiD proteins (MiD49 and MiD51), which work with Drp1 to promote fission independently of Fis1 and MFF [14]. Although there is conflicting evidence on the role of these adaptor proteins in mitochondrial dynamics, it appears that they work together to promote fission [14]. As discussed below, our data suggest that Fis/Drp1 interaction mediates pathological fission whereas interaction between Drp1 and MFF or MiD proteins may be involved in physiological fission. We found that an inhibitor of Drp1 and Fis1 interaction had no effect in healthy animals but reduced heart failure development after myocardial infarction [15].

To maintain cell integrity, there needs to be a balance between mitochondrial fission and fusion. Fusion requires both the outer and inner membranes of two mitochondria to fuse. At the outer mitochondrial membrane, this process is directed by two other members of the large GTPase dynamin family, mitofusin 1 (Mfn1) and mitofusin 2 (Mfn2) [16]. In addition to its role in fusion, Mfn2 links endoplasmic reticulum to mitochondria [17]. Opa1, another member of the dynamin family (named Opa for the mutation identified in dominant optic atrophy) [18], mediates inner membrane fusion as well as cristae remodeling independently of mitochondrial fusion [19].

Together, Drp1, Mfn1, Mfn2, and Opa1 with their respective (known and unknown) adaptor/interacting proteins work to maintain a proper balance between fission and fusion (Table 1). Loss of mitochondrial fission-fusion balance is associated with a number of diseases, predominantly neurodegenerative diseases and cardiovascular diseases [1, 20]. Here, we discuss a potential therapeutic approach to rescue the heart from mitochondrial damage during ischemia-reperfusion injury and heart failure, focusing on compounds that regulate mitochondrial dynamics and quality control.

**Table 1:** List of mitochondrial dynamic proteins

Proteins	Location at mitochondria	Function	Interacting protein	Properties
Opa1	Inner membrane	Inner membrane fusion	Not known	GTPase activity
Fis1	Outer membrane	Outer membrane fission	Drp1	adaptor protein
Drp1	Outer membrane	Outer membrane fission	Fis1	GTPase activity
Mff	Outer membrane	Outer membrane fission	Drp1	adaptor protein
MiD49/51	Outer membrane	Outer membrane fission	Drp1	adaptor protein
Mfn1	Outer membrane	Outer membrane fusion	Mfn2	GTPase activity
Mfn2	Outer membrane	Outer membrane fusion; link mitochondria to ER; autophagy	Parkin; Mfn1	GTPase activity

## Cardiovascular diseases

Cardiovascular diseases, including coronary artery disease, hypertension, ventricular hypertrophy, myocardial infarction, and heart failure are leading causes of death worldwide. The establishment and progression of these diseases involve multiple processes including over-activation of the sympathetic nervous system and the renin-angiotensin-aldosterone system, as well as inflammation [21]. Mitochondria have been considered key sensors and effectors of cardiac pathophysiology. In addition to their ability to produce energy, cardiac mitochondria directly regulate several other intracellular processes such as calcium homeostasis, apoptosis, nuclear gene expression, ion gradients, redox potential of the cells, and contractility; balanced mitochondrial fission/fusion are critical for these functions [15, 22–25]. Therefore, regulating mitochondrial fusion- and fission-related proteins has become an attractive target for novel therapies for cardiac diseases.

## Mitochondrial dynamics in cardiac diseases

### Regulation of mitochondrial fission

**Fig. 1:**
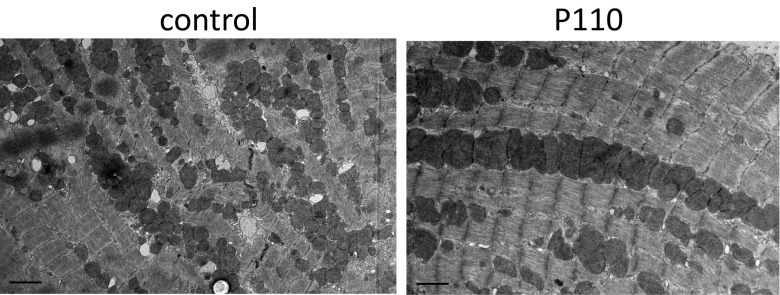
Representative TEM micrograph of a rat heart subjected to ischemia and reperfusion (IR) using an ex vivo model of myocardial infarction. Treatment with the Drp1 inhibitory peptide, P110 (*right*), blocked excessive mitochondrial fission observed in hearts subjected to IR in the presence of control peptide. *Bar* = 2 μm

Mitochondrial dysfunction plays a key role in ischemia and reperfusion (IR) injury, cardiomyopathy, and heart failure [1, 26, 27]. Inhibition of Drp1/Fis1 interaction in cultured murine cardiac myocytes and in whole rat heart models of IR reduced excessive mitochondrial fission and heart damage [28]. Mitochondrial swelling and fragmentation were accompanied by dephosphorylation of serine 637 of Drp1 by calcineurin. The fission inhibitors, Mdivi-1, Drp1 siRNA, calcineurin inhibitor, or therapeutic hypothermia all reversed these pathologies in these IR models [28]. Treatment with Mdivi-1, a Drp1 inhibitor, prior to ischemia also reduced mitochondrial damage and myocardial infarct size in mice subjected to transient coronary artery occlusion [29]. Drp1 inhibition by the heptapeptide inhibitor, P110 [15, 30], inhibited IR-induced excessive mitochondrial fission, as shown by electron microscopy (Fig. 1) and analysis of mitochondrial size by fluorescence-activated cell sorting (FACS) [15]. We also demonstrated that a single dose of P110 peptide at reperfusion after transient coronary artery occlusion inhibited mitochondrial fragmentation, increased ATP levels and mitochondrial size, and improved cardiac functions when measured 3 weeks after the occlusion [15]. Importantly, in contrast to Mdivi-1, P110 had no effect on the basal activity of Drp1. This may be due to the selectivity of P110 for the Drp1-Fis1 interaction and the lack of any effect on Drp1 binding to other adaptor proteins, such as MFF. Since one single dose of P110 was sufficient to reduce heart dysfunction even 3 weeks after myocardial infarction (MI), inhibiting fragmentation at the onset of the injury was sufficient for prolonged effect. Together, inhibition of Drp1, specifically targeting Drp1/Fis1 interaction, appears to have therapeutic potential in preventing MI-induced cardiac injury and subsequent heart failure development.

Drp1 undergoes modifications other than dephosphorylation, which can also induce fission. Recently, sumoylation/desumoylation of Drp1 was also found to regulate mitochondrial function [31]. Sumoylation protected Drp1 from degradation, and the subsequent increase in Drp1 activity may contribute to cardiomyopathy and heart failure [32]. Overexpression of a SUMO isopeptidase led to increased mitochondrial size as well as altered mitochondrial morphology and mitochondrial dysfunction in mouse hearts [32]. Furthermore, hearts overexpressing adenovirus dominant-negative Drp1 (Drp1K38A) exhibited a lower oxygen consumption rate and improved mitochondrial membrane potential accompanied by increased mitochondrial fusion, which led to cardioprotection after IR [29, 33]. Those elegant reports using viral infection demonstrated that modulating Drp1 might be a good therapeutic target for cardioprotection. However, viral infection has limited therapeutic use in humans, and pharmacological agents targeting this process are still unavailable.

In addition, the antiapoptotic serine/threonine kinase Pim-1 mediated Drp1 phosphorylation and sequestration in the cytosol. Overexpression of Pim-1 caused a reduction in Drp1 levels and preservation of the mitochondrial network [34, 35]. Therefore, inhibitors of some kinases (e.g., Pim-1) and phosphatases (e.g., calcineurin) may improve mitochondrial function and therefore protect the myocardium from both acute and chronic insults. However, chronic use of kinase or phosphatase inhibitors is probably impractical because of their prominent role in signaling pathways other than those modulating mitochondrial function.

Together, these studies demonstrate that inhibition of excessive mitochondrial fission improves cardiac health. However, because physiological fission is required to maintain healthy mitochondria in tissue with high energy demand, such as the heart, the inhibitors used need to be highly specific for pathological fission.

### Regulation of mitochondrial fusion and trans-organelle linkage

Mfn1, Mfn2, and OPA1 are the main proteins involved in mitochondrial fusion. Oxidative stress-mediated downregulation of Mfn1 resulted in accumulation of fragmented mitochondria and apoptosis in neonatal rat cardiomyocytes [36]. Knockdown of Mfn1 aggravated the above damage, whereas Mfn1 overexpression prevented these H_2_O_2_-related injuries [36]. Unexpectedly, hearts from mice with cardiomyocyte-specific deletion of Mfn1 accumulated fragmented mitochondria that exhibited preserved function, resistance to oxidative stress, and increased calcium-induced permeability [37]. However, there are no data on the susceptibility of these mice to cardiomyopathy development upon stress. The explanation for the differing effects of Mfn1 depletion in isolated neonatal cardiomyocytes and adult hearts may reflect differences in mitochondrial architecture; under basal conditions, mitochondrial fusion is spatially limited by the tight organization of the contractile elements in adult cardiomyocytes, but not in neonatal cultured myocytes.

Mfn2 knockout mice exhibited increased mitochondrial size, dissipation of mitochondrial inner membrane potential, and increased ROS generation; cardiac hypertrophy and ventricular dysfunction occurred in older Mfn2 knockout mice [38–40]. The main role of Mfn2 in maintaining ventricular function and morphology is likely related to its ability to tether the sarcoplasmic reticulum to the mitochondria [17]. Ablation of cardiac Mfn2, but not Mfn1, decreased the contact length between sarcoplasmic reticulum (SR) and mitochondria, increased SR calcium content, and disrupted calcium handling in isolated cardiac myocytes [41, 42]. In addition to its role in regulating SR-mitochondrial tethering, Mfn2 plays a crucial role in mitochondrial elimination through the recruitment of PTEN-induced putative kinase 1 (PINK1)-Parkin to damaged mitochondria [38], which complicates the interpretation of the earlier studies regarding Mfns.

Combined Mfn1 and Mfn2 ablation in mouse hearts resulted in accumulation of dysfunctional and fragmented mitochondria and led to lethal heart failure at approximately 8 weeks after Mfn1/Mfn2 ablation [41]. Furthermore, cardiomyocyte expression of human Mfn1 or Mfn2 rescued the cardiomyopathy observed in *Drosophila* with no expression of mitochondrial assembly regulatory factor (MARF), a *Drosophila* ortholog of mammalian Mfns [43]. These results demonstrated that proper fusion/fission balance is essential for maintaining cardiac functions [17]. Clearly, Mfn1 and 2 have distinct roles: a single Mfn1 allele and no Mfn2 expression in mice did not affect baseline cardiac function, whereas mice with a single cardiac Mfn2 allele and no Mfn1 expression developed cardiomyopathy at 8 weeks of age [17].

Finally, Opa1, the third large GTPase that is involved in mitochondrial fusion, also plays a key role in cardiac physiology. Posttranslational proteolytic processing of Opa1, generating long Opa1 isoforms (anchored to the inner mitochondrial membrane) and short isoforms (found in the intermembrane space), is important in controlling both the fusion of the inner mitochondrial membrane and organization of the cristae structure. Loss of Opa1 led to mitochondrial fragmentation and aberration in cristae structure [19]. Heterozygotic Opa1^+/−^ mice accumulated fragmented and dysfunctional mitochondria and exhibited loss of mitochondrial DNA stability and increased ROS generation in the heart. The corresponding cardiomyocytes displayed reduced calcium transients, impaired contractility, and increased susceptibility to IR-induced injury [44]. Therefore, along with Drp1, at least two other enzymes that control mitochondrial dynamics, Mfn1 and Opa1, are essential for maintaining mitochondrial integrity and cardiac functions; pharmacological agents that activate Mfn1 and Opa1 may also have cardioprotective effects as long as they will selectively affect pathological fusion.

Obesity and diabetes are independent risk factors for cardiovascular disease development. Ventricular dysfunction and hypoxic insult as well as reduced cardiomyocyte contractile properties are closely related to mitochondrial dysfunction and increased oxidative stress in diabetic cardiomyopathy and obesity in humans [45, 46]. These metabolic disorders are associated with the disruption of cardiac mitochondrial fusion-fission balance. Worsening of myocardial contractile properties during transition from obesity to diabetes was reported in humans to correlate with reduced cardiac Mfn1 levels, accumulation of fragmented mitochondria, and metabolic disruption [47]. Of interest, decreased cardiac Atg5 protein levels (a protein required for autophagy; see below) were also observed in diabetic patients but not in obese patients, suggesting a role of mitochondrial fusion-fission balance (mitochondrial dynamics) and clearance in diabetic cardiomyopathy. An imbalance in mitochondrial dynamics might contribute to the establishment and/or progression of obesity- and diabetes-related cardiomyopathy.

Accumulation of fragmented dysfunctional mitochondria has been reported in myocardial infarction-induced heart failure model in animals [48, 49]. Furthermore, failing human hearts have decreased Opa1 levels and increased levels of Mfn1 and Mfn2 to likely compensate for the reduction in Opa1 [49]. Accumulation of nonfunctional Mfn may be a consequence of impaired proteasomal activity observed in failing human hearts [50]. The role of mitochondrial dynamic imbalance in heart failure development and progression in humans remains to be elucidated. However, it appears from the works cited above that controlling mitochondrial dynamics should improve cardiac health.

## Mitophagy in the heart

To limit the damage induced by dysfunctional mitochondria following IR injury, the heart activates a protective mechanism by which damaged mitochondria (containing damaged/oxidized proteins and damaged mitochondrial DNA) are eliminated through a process of mitochondrial autophagy and mitophagy. Autophagy is an essential catabolic process involving degradation of unnecessary or dysfunctional cellular components (including mitochondria) by lysosomes. Under normal conditions, autophagy is kept at a basal level to maintain cellular homeostasis and preserve cell integrity by eliminating long-lived, overproduced, and aggregation-prone proteins or dysfunctional organelles such as damaged mitochondria. Much has been written about the role of autophagy induced by starvation, but this aspect of autophagy appears to be less relevant to the heart. Cardiac autophagy that is triggered by stress, such as by IR, promotes cell survival [51]. Particularly, proper elimination of damaged mitochondria under such conditions is important to protect cells against the release of proapoptotic proteins, such as Bcl-2, and the production of excessive mitochondrial ROS [52–54]. Therefore, autophagy contributes to the maintenance of quantity and quality of cardiac mitochondria.

**Fig. 2:**
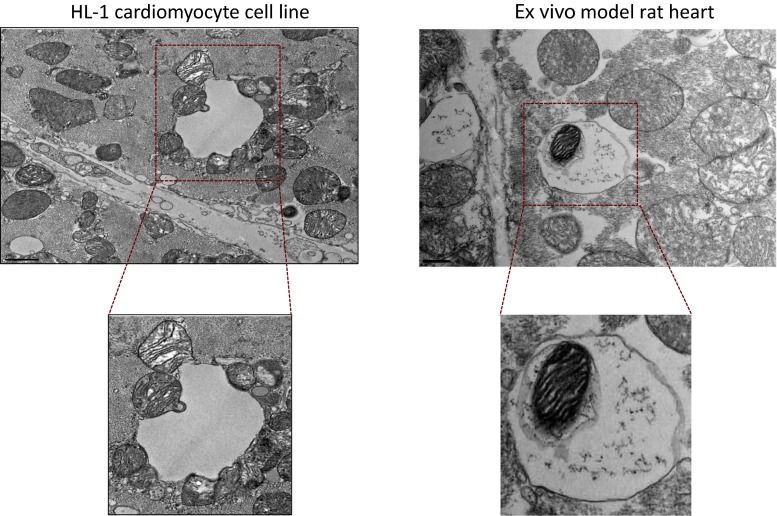
Representative TEM micrograph. HL-1 cardiomyocyte cell subjected to ischemia and reperfusion (IR) (*left*). Shown are mitochondria undergoing macroautophagy and micromitophagy. Mitochondria directly fused with the lysosomal membrane for degradation represent an example of microautophagy. A mitochondrion inside a lysosome is also seen on the *right*, providing an example of mitophagy in an ex vivo model of rat myocardial infarction. *Left panel*, *bar* = 1 μm; *right panel*, *bar* = 0.5 μm

**Fig. 3:**
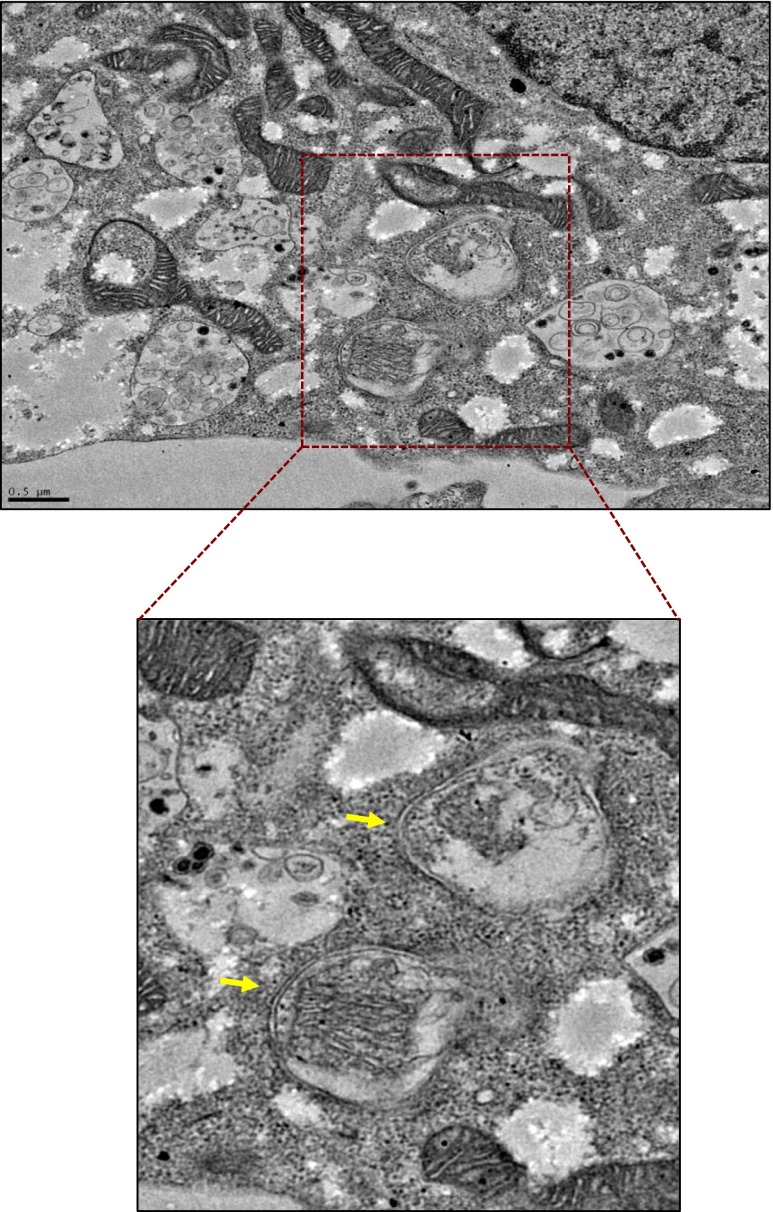
Representative TEM micrograph of intact HL-1 cardiomyocyte cell following IR-induced injury. An example of macroautophagy: mitochondria are encapsulated in double-membrane vesicles (autophagosomes; *arrows*), which are ultimately targeted to lysosomes for degradation. *Bar* = 0.5 μm

Depending on how mitochondria are delivered to lysosomes, mitochondrial elimination occurs by two pathways—macromitophagy and micromitophagy (Figs. 2 and 3). Macromitophagy is characterized by sequestration of mitochondria into double-membrane structures, called autophagosomes, which are sequentially fused with lysosomes where the mitochondria are degraded. A number of molecular pathways regulate macromitophagy. PTEN-induced putative kinase 1 (PINK1) accumulates at the outer mitochondrial membrane of damaged mitochondria and recruits the ubiquitin ligase E3-associated protein, Parkin. Parkin induces ubiquitination of mitochondrial proteins and degradation of damaged mitochondria in lysosomes. This process is further regulated by voltage-dependent anion channels (VDACs), BECN1-regulated autophagy protein 1 (AMBRA1), or p62/SQSTM1 (sequestosome 1) complexes [55–58]. In addition, the serine threonine kinase, Ulk1, and FUN14 domain-containing 1 (FUNDC1) induce Pink1/Parkin-independent macromitophagy in mouse embryonic fibroblast (MEF) cells exposed to hypoxia [59]. Furthermore, NIP1-like protein X, Nix (also called BNip3L), and BNip3, which might have multiple effects on mitochondria, have also been found to induce tethering of damaged mitochondria to lysosomes for autophagy and thus to be cardiac protective [60].

Micromitophagy, where mitochondria are directly engulfed by lysosomes also occurs following cell stress [61, 62]. Recently, our group showed that micromitophagy is independent of the macromitophagy pathway [63]. Using cell cultures and whole heart, we found that IR-induced injury stimulated the association of inactivated glyceraldehyde-3-phosphate dehydrogenase (GAPDH) with mitochondria, which induced direct fusion of damaged mitochondria into lysosomal-like (likely early endosomes) structures for removal [63]. As oxidative injury increased, protein kinase C delta (PKCδ) inhibited GAPDH-induced micromitophagy by phosphorylating GAPDH at Thr246. PKCδ-induced GAPDH phosphorylation promoted the accumulation of damaged mitochondria, leading to mitochondrial-induced cell death by apoptosis [63]. Inhibition of the macroautophagy machinery did not affect this GAPDH-dependent elimination of damaged mitochondria by micromitophagy [63].

How are mitochondrial fission and mitophagy linked? Mitochondrial fission appears to be required for mitophagy under basal growth condition and during nutrient starvation in MEF cells [61, 62]. The synergistic role of Drp1 and Parkin in mitochondrial homeostasis in cardiomyocytes supports a link between mitochondrial fission and mitophagy [64]. In a mouse model of heart failure, inhibition of excessive mitochondrial fission during IR inhibited mitophagy, indicating coordination between these two mechanisms [65]. Furthermore, mild oxidative stress induced both mitochondrial fragmentation and mitophagy in non-cardiac cells [66]. Together, the interplay between mitochondrial fission and mitophagy seems to depend on the conditions in which the mitochondria become damaged and the amount (or degree) of stress. In addition to fission, as described earlier, mitofusin 2 plays an essential role in mitophagic mitochondrial quality control, anchoring Parkin to damaged cardiomyocytes mitochondria [38, 67]. A recent review described the crosstalk between the quality control machineries for cardioprotection [67], suggesting a link between mitochondrial regeneration by fusion and mitophagy. Regardless of whether mitophagy depends on mitochondrial fission and fusion, drugs that increase the selective removal of damaged mitochondria by mitophagy are expected to be cardioprotective by decreasing oxidative stress and apoptosis induced by dysfunctional mitochondria.

## Mitophagy in cardiac diseases

Basal levels of mitophagy or macroautophagy under mild stress were found to be important; they preserve myocardial (or cellular) homeostasis and thus maintain normal cardiac functions. Cardiac-specific Atg5-deficient mice exhibited cardiac dysfunction 1 week after subjecting the heart to pressure overload [68], whereas overexpression of autophagic genes ameliorated cardiomyopathy [69]. Parkin-deficient mice showed decreased survival with increased vulnerability to myocardial infarction [70, 71]. The disruption of PINK1/Parkin interaction further impaired mitochondrial function and mitophagy in aged hearts, leading to cardiac dysfunction [72]. Beclin, a mammalian ortholog of the yeast autophagy-related gene Atg6, regulates autophagy. Beclin1 knockdown by RNAi reduced autophagic flux following 2 h ischemia and 5 h reperfusion-induced injury in cardiomyocytes. The authors demonstrated a significantly increased apoptosis mediated by an increase in Bax activation, a member of the BCL2 gene family [73]. Together, these studies support protective and adaptive functions of mitophagy (by micro- or macro-autophagy) to promote survival.

However, in response to more severe oxidative stress, such as that induced by prolonged hypoxia followed by reperfusion, cells are not rescued by mitophagy. Apoptosis is increased either because the mechanisms involved in mitophagy are impaired (e.g., because of ATP shortage due to a high number of impaired mitochondria) or because the process of mitophagy cannot keep up with the number of damaged mitochondria, thus, leading to insufficient lysosomal elimination of the damaged mitochondria [74]. In addition, upregulated mitophagy machinery may promote cell death by clearing healthy mitochondria [75]. Unlike the results in primary cardiomyocytes as described earlier [73], in Beclin 1^+/−^ mice subjected to IR, the number of autophagosomes and the size of myocardial infarction were significantly reduced, suggesting that induction or activation of autophagy can be detrimental [76]. Another study showed that IR-induced upregulation of Beclin 1 was accompanied by a rapid decline in LAMP2, a protein important for the fusion of macroautophagosomes with lysosomes, provoking cardiomyocyte death [77]. Furthermore, inhibition of autophagy by downregulating Beclin 1 or the use of 3-methyladenine (a macroautophagy inhibitor) was protective when neonatal cardiomyocytes were exposed to simulated IR or to H_2_O_2_-induced injury [76, 78]. Taken together, these results suggest that the protective effect of autophagy remains controversial. Therefore, pharmacological upregulation of autophagy (e.g., with rapamycin, chloramphenicol succinate, or SAHA, an HDAC inhibitor) or GAPDH-driven mitophagy (e.g., inhibiting PKCδ translocation to mitochondria) may enhance the clearance of damaged mitochondria and thus prevent the onset of cell death following IR-induced injury [63, 73, 79, 80]. An understanding of the balance between cardioprotective mitophagy and cell death will provide useful insights into developing new therapeutic strategies for cardiovascular diseases. However, until the role of macroautophagy and micromitophagy in stressed mycardium is determined, the use of inhibitors or activators of these processes in humans may be premature.

## Conclusion

The central role of mitochondria in the health of the myocardium has been recently recognized. As discussed above, the machineries regulating mitochondrial fusion and fission and removal of damaged mitochondria by autophagy are potential novel therapeutic targets for cardiovascular disease. However, further research into the critical molecular events that should be regulated is needed to develop the optimal pharmacological strategy to treat these diseases.
